# Intraarticular injection of processed lipoaspirate cells has anti-inflammatory and analgesic effects but does not improve degenerative changes in murine monoiodoacetate-induced osteoarthritis

**DOI:** 10.1186/s12891-019-2710-1

**Published:** 2019-07-19

**Authors:** Takumi Sakamoto, Tsuyoshi Miyazaki, Shuji Watanabe, Ai Takahashi, Kazuya Honjoh, Hideaki Nakajima, Hisashi Oki, Yasuo Kokubo, Akihiko Matsumine

**Affiliations:** Department of Orthopaedics and Rehabilitation Medicine, Faculty of Medical Sciences, University of Fukui, Matsuoka Shimoaizuki 23-3, Eiheiji, Fukui, 910-1193 Japan

**Keywords:** Processed lipoaspirate (PLA) cells, Mesenchymal stem cells (MSCs), Adipose derived-mesenchymal stem cells (AD-MSCs), Intra-articular injection, Osteoarthritis (OA), Monoiodoacetate (MIA), Sprague-Dawley rats, Dorsal root ganglion (DRG), Calcitonin gene-related peptide (CGRP), Substance P (SP)

## Abstract

**Background:**

Previous basic research and clinical studies examined the effects of mesenchymal stem cells (MSCs) on regeneration and maintenance of articular cartilage. However, our pilot study suggested that MSCs are more effective at suppressing inflammation and pain rather than promoting cartilage regeneration in osteoarthritis. Adipose tissue is considered a useful source of MSCs; it can be harvested easily in larger quantities compared with the bone marrow. The present study was designed to evaluate the anti-inflammatory, analgesic, and regenerative effects of intra-articularly injected processed lipoaspirate (PLA) cells (containing adipose-derived MSCs) on degenerative cartilage in a rat osteoarthritis model.

**Methods:**

PLA cells were isolated from subcutaneous adipose tissue of 12-week-old female Sprague-Dawley rats. Osteoarthritis was induced by injection of monoiodoacetate (MIA). Each rat received 1 × 10^6^ MSCs into the joint at day 7 (early injection group) and day 14 (late injection group) post-MIA injection. At 7, 14, 21 days after MIA administration, pain was assessed by immunostaining and western blotting of dorsal root ganglion (DRG). Cartilage quality was assessed macroscopically and by safranin-O and H&E staining, and joint inflammation was assessed by western blotting of the synovium.

**Results:**

The early injection group showed less cartilage degradation, whereas the late injection group showed cartilage damage similar to untreated OA group. The relative expression level of CGRP protein in DRG neurons was significantly lower in the two treatment groups, compared with the untreated group.

**Conclusions:**

Intra-articular injection of PLA cells prevented degenerative changes in the early injection group, but had little effect in promoting cartilage repair in the late injection group. Interestingly, intra-articular injection of PLA cells resulted in suppression of inflammation and pain in both OA groups. Further studies are needed to determine the long-term effects of intra-articular injection of PLA cells in osteoarthritis.

## Background

Osteoarthritis (OA) of the knee is common and one of the major causes of joint dysfunction and physical disability in the elderly [1]. OA of the knee is associated with knee-related disabling symptoms in about 10% of UK men and women aged more than 55 years, and about 25% of these patients suffer debilitating disability [2]. Muraki et al. [3] reported that 49.5% of elderly Japanese had radiographically-evident bilateral OA of the knee (Kellgren and Lawrence (K/L) grade ≥ 2). Furthermore, the estimated prevalence of knee OA is 42.6 and 62.4% in Japanese men and women, respectively [4], and 19.7% of residents of Framingham, Massachusetts, USA [5].

Although total knee arthroplasty and tibial osteotomy are currently performed worldwide to eliminate knee pain and improve joint function, knee OA is often managed conservatively with medications, intra-articular injections, brace, fomentation and physiotherapy. In this context, it is desirable to develop effective therapies for knee OA that are less invasive, inexpensive, safe, and produce rapid and long-lasting effects. However, cartilage tissues have limited intrinsic capacity for repair and no medications have yet been developed that can achieve durable modification of OA progression.

Mesenchymal stem cells (MSCs) are promising for use in cartilage regeneration and treatment of OA since these cells show chondrogenic potential and ability to create extracellular matrix [6]. In the field of rheumatology, MSCs obtained from the bone marrow (BM-MSCs) are sometime injected into the cartilage or bone using three-dimensional scaffold fixed to the defective area of the joint. Recent studies reported satisfactory survival rates and significant improvement in function and pain relief after autologous chondrocyte implantation in adolescent patients over long-term follow-up [7]. However, a technically demanding surgical procedure is required for successful application of the BM-MSCs via the scaffold. Recently, direct intraarticular injection of BM-MSCs has been reported and the clinical usefulness of several procedures has been tested for clinical application of BM-MSCs for articular cartilage repair [8]. Animal studies using cell tracking have demonstrated limited cartilage formation following chondrogenic differentiation of BM-MSCs [9–11], with the injected cells mainly being localized in other parts of the joint, including the synovium.

Experimental evidence suggests that injection of BM-MSCs into OA joints is followed by decrease in synovial fluid levels of inflammatory cytokines [12]. These findings suggest the potential dual role of BM-MSCs in the treatment of OA, i.e., these cells do not only promote regeneration of damaged cartilage but also contribute to joint homeostasis. In previous animal studies, loading of the affected limb showed a significant increase 4 weeks after injection of BM-MSCs, suggesting alleviation of pain, although such cell therapy had no significant effect on joint damage and synovial inflammation [13].

In addition to the above characteristics, BM-MSCs also have immunomodulatory and trophic effects related to the production of anti-inflammatory and growth factors [14], which probably improve the inflammatory and catabolic aspects of OA. However, there are no clinical or experimental studies on the effects of intraarticular injection of adipose-derived mesenchymal stromal cells (AD-MSCs) in the prevention of degenerative changes of OA cartilage, including their anti-inflammatory and analgesic effects. In this regard, Zuk and colleagues were the first group to describe the isolation of AD-MSCs from human subcutaneous fat and that these stem cells can differentiation into different types of tissues [15]. Recent studies have focused on the properties of adipose tissue and potential use as an alternative to bone marrow cells. Several advantages of AD-MSCs have been described, relative to BM-MSCs, including ease of harvesting these cells under local anesthesia and ability to obtain larger quantities of stem cells than BM-MSCs [16]. In this regard, one study is estimated the preparation of 5 × 10^3^ AD-MSCs from only approximately 1 g of adipose tissue, which is 500-fold greater than those obtained from the bone marrow [17]. Other advantages of AD-MSCs is their higher tolerance to hypoxia, serum-free conditions and oxidative stress compared with BM-MSCs under in vitro conditions. Moreover, the survival rate of AD-MSCs is higher than that of BM-MSCs when transplanted into sites of severe spinal cord injury in vivo [18].

The present study was designed to evaluate the effects of intraarticular injection of processed lipoaspirate (PLA) cells (known as multipotent stem cells [15]) into the knee joint, especially on inflammation, pain and cartilage damage in an animal model of monoiodoacetate (MIA)-induced arthritis.

## Methods

### Experimental animals

All experiments followed the guidelines for the study of pain in awake animals established by the International Association for the Study of Pain [19] as well as the ARRIVE guidelines. All animal care and experiments were conducted in accordance with the institutional guidelines of our Animal Committee and the study protocol was approved by the Animal Care Ethics Committee for Experimental Studies of Fukui University.

Experiments were conducted on 90 female Sprague-Dawley rats (Clea, Tokyo, Japan), aged 3–4 months with a mean body weight of 282.2 ± 7.3 g (±standard deviation). The animals were housed under conditions of controlled temperature (23 ± 1 °C) and had free access to water and food.

### Preparation of processed lipoaspirate (PLA) cells

PLA cells were isolated from 12-week-old female Sprague-Dawley rats. Briefly, subcutaneous fat was harvested under general anesthesia. The collected tissue was minced using a razor blade, digested with 0.075% collagenase type I (Sigma-Aldrich, St. Louis, MO) at 37 °C for 50 min, and the resultant cell suspension was filtered through 70 μm filter, then centrifuged (250×*g*) for 5 min. The obtained cells were washed thrice in phosphate-buffered saline (PBS; Gibco BRL, Grand Island, NY), then resuspended in Dulbecco’s modified Eagle’s medium (DMEM; Gibco) containing 10% fetal bovine serum (FBS; Gibco). The cell cultures were kept under 5% CO_2_ in a feedback automatic incubator set at 37.5 °C to maintain 80–90% confluence. Finally, 0.025% trypsin/EDTA (Gibco) solution was used for cell digestion before cell passage, as described previously [15].

### Flow cytometry

Flow cytometric analysis was performed to determine the surface expression of specific proteins on PLA cells (*n* = 5). After the third passage, the cells were trypsinized and single-cell suspensions were obtained and immunostained on ice for 45 min with PE-CD29, −CD31 and -CD90.1 (each at 0.25 μg/100 μl) fluorochrome-conjugated anti-rat antibodies (BD Biosciences). For control antibodies, we used the corresponding mouse isotype antibodies (dilution, 1:5; Santa Cruz Biotechnology, Santa Cruz, CA). A fluorescence activated cell sorter (FACSCanto II; BD Biosciences, Franklin Lakes, NJ) was used for cell analysis, using the protocol provided by the manufacturer. Data for 10,000 gated cell events were processed by proprietary software (FACSDiva; BD Biosciences) and the antigen expression rate was calculated.

### Intraarticular injection of MIA and neurotracer

On day 0, Sprague-Dawley rats were anesthetized with isoflurane and injected intraarticularly with MIA (2 mg, Sigma-Aldrich) and a retrograde neurotracer (2% fluoro-gold in 25 μl of 0.9% saline; Fluorochrome, Denver, CO). Injection was performed through the infrapatellar ligament using a 27G needle with the knee flexed at 90°. Then the animals were allowed to recover and kept in cages under a 12–12 h light–dark cycle.

### Intraarticular injection of PLA cells

**Fig. 1:**
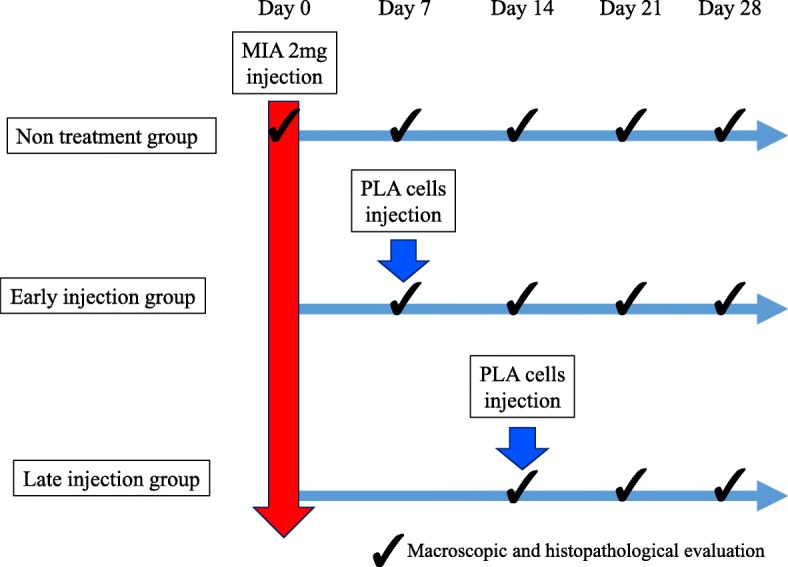
On day 0, Sprague-Dawley rats were anesthetized with isoflurane and injected intraarticularly with 2 mg monoiodoacetate (MIA) and a retrograde neurotracer (2% fluoro-gold in 25 μl of 0.9% saline). They were randomly separated into three groups of non-treatment group, early injection group, and late injection group. In each animal, 1 × 10^6^ PLA cells were injected into the right knee while saline was injected in the left knee as a control. PLA cells were injected on day 7 (early injection group) or 14 (late injection group) after administration of MIA. Rats were euthanized by CO_2_ at 7, 14, 21 days after MIA administration, and the knee joints and spines were harvested for examination

Sprague-Dawley rats were randomly separated into three groups of non-treatment group (*n* = 40), early injection group (*n* = 30), and late injection group (*n* = 20). Each rat was first anesthetized with isoflurane (Forane®; Abbot, Tokyo), and then PLA cells were injected at a dose of 1 × 10^6^ cells per joint into the right knee while saline was injected in the left knee as a control. This procedure was performed on day 7 (early injection group) or 14 (late injection group) after administration of MIA. Rats were euthanized by CO_2_ at 7, 14, 21 days after MIA administration, and the knee joints and spines were harvested for analysis (Fig. 1).

### Tissue preparation

Under deep anesthesia, the peri-articular soft tissues (including the synovium and joint capsule) were resected along with the joint cartilage. Then the rats were perfused transcardially with 0.9% saline, followed by 4% paraformaldehyde in phosphate buffer. MIA-treated joints were prepared for histopathological examination, while the spinal dorsal root ganglia (DRGs) were prepared for immunohistological assessment.

### Macroscopic and histopathological evaluation of the knee joint

Next, we assessed the cartilage quality by assigning a score that ranged from 0 to 5 using the grading system described by Yoshioka et al. [20]. Briefly, the following criteria were applied to assess the morphological changes of the femoral condyles after the application of India ink. Grade 1 (intact surface): the condylar surface appeared normal and did not retain any ink. Grade 2 (minimal fibrillation): the condylar surface appeared normal before staining, but retained India ink as elongated specks or light gray patches. Grade 3 (overt fibrillation): the condylar cartilage appeared velvety and retained black patches of ink. Grade 4 (erosion): evidence of cartilage loss with exposure of the underlying bone.

The resected limbs were cut at the midfemoral and midtibial levels and immersed in 4% buffered paraformaldehyde fixative for 1 week at 4 °C. Then the samples were decalcified in KCX solution (Falma, Japan), dehydrated and embedded in paraffin, cut into 10 mm sections, and stained with safranin-O and hematoxylin and eosin stain for light microscopy.

### Immunostaining of DRG specimens

DRG specimens were harvested and processed at 7, 14, and 21 days after induction of OA (i.e., injection of MIA). The specimens were treated overnight with buffered paraformaldehyde fixative at 4 °C and then with PBS containing 20% sucrose for 20 h at 4 °C, then frozen in liquid nitrogen, before sectioning into 10-μm thick sections using a cryostat. Sections were incubated with rabbit anti-calcitonin gene-related peptide (CGRP) antibody and goat anti-SP antibody for 24 h at 4 °C, followed by incubation for 2 h at 4 °C with Alexa A11034 anti-rabbit IgG (to detect CGRP immunoreactivity) or Alexa A11079 anti-goat IgG (to detect SP immunoreactivity). The sections were rinsed thrice in PBS after each step and the immunostained sections were assessed by fluorescence microscopy by an investigator who was blinded to the treatment. The number of FG-labeled DRG neurons with CGRP or SP immunoreactivity was counted and calculated as a percentage of the total number of FG-labeled DRG neurons in each section.

### Western blot analysis of DRG and knee joint synovium

Cells were rinsed in a phosphate buffer and lysed in RIPA lysis buffer to isolate proteins from PLA cells and knee joint synovium, which were then collected by centrifugation. The membrane was immersed for 1 min in ECL Advance Western Blot Detection kit (GE Healthcare, Buckinghamshire, UK) and X-ray filmed to visualize peroxidase activity and to determine the level of each protein. The intensity of each band was expressed relative to that of β-actin (1:2000, Abcam, Cambridge, UK). Kaleidoscope Prestained Standards (Bio-Rad Laboratories, Hercules, CA) were used as molecular weight markers.

### Statistical analysis

All experiments and analyses were performed with at least 5 animals per group. Data were reported as mean ± SD. Differences between groups were assessed by one-way ANOVA. *P* < 0.05 denoted the presence of significant difference with Tukey’s post-hoc analysis.

## Results

### Characterization of PLA cells

**Fig. 2:**
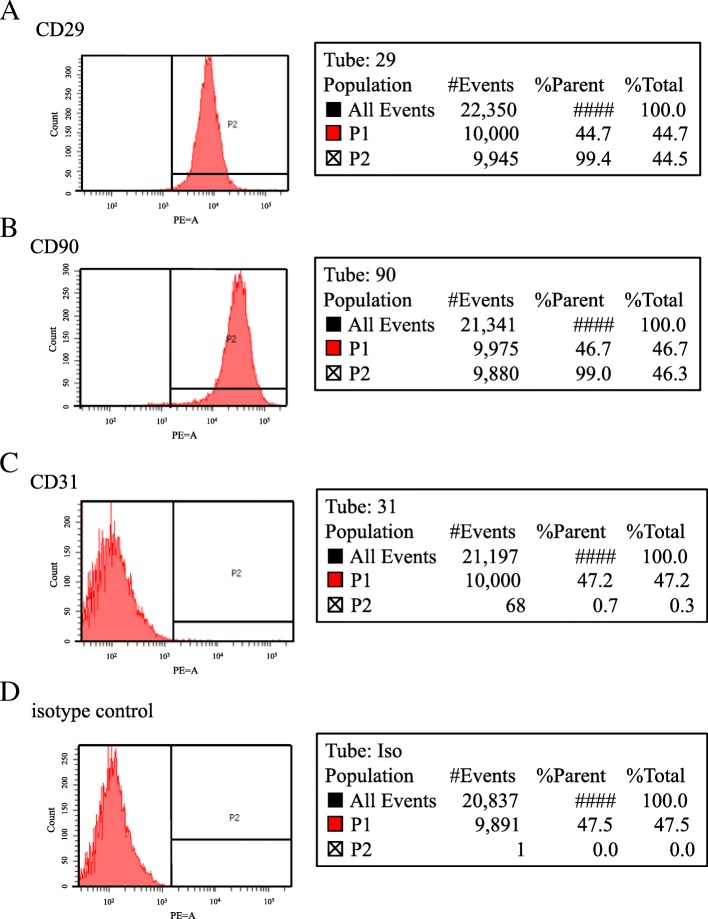
The phenotypic profile of rat PLA cells, determined by flow cytometry. The rat PLA cells were 99.4% positive for CD29 (**a**), 99.0% positive for CD90 (**b**), 99.7% negative for CD31 (**c**), and 100% negative for isotype control (**d**)

PLA cells were isolated from the subcutaneous adipose tissue and expanded to form confluent cultures of adherent cells with fibroblastic morphology. Flow cytometry was used to determine the phenotypic profile of the rat PLA cells: 99.4% of these cells were positive for CD29 (Fig. 2a), 99.0% positive for CD90 (Fig. 2b), 99.7% negative for CD31 (Fig. 2c), and 100% negative for isotype control (Fig. 2d).

### Effect of intraarticular injection of PLA cells on cartilage degradation

**Fig. 3:**
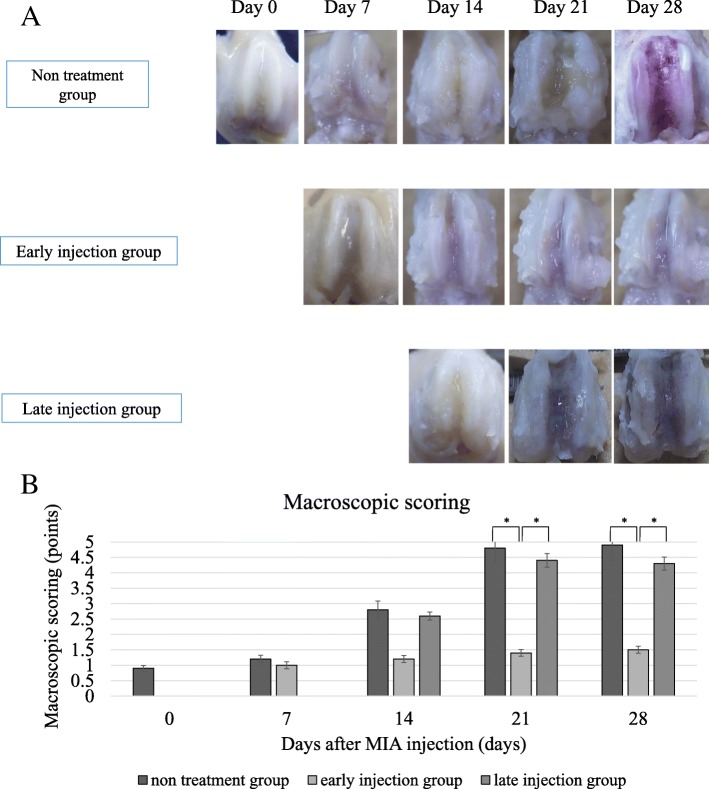
Representative macroscopic features of the femoral condyle cartilage. In the untreated OA group and late injection group, cartilage erosion was observed at day 14, and bone destruction was evident at day 21 and progressed at day 28. In the late injection group, the cartilage surface was still glossy at day 28 (**a**). The scores were 2.9, 4.4, and 4.3 at days 14, 21, and 28 after injection, respectively. In the early injection group, the scores of the treated knees were lower than the control knees. Data are mean ± SD, *P <* 0.05, by one-way ANOVA*,* with Tukey’s post-hoc analysis (**b**)

**Fig. 4:**
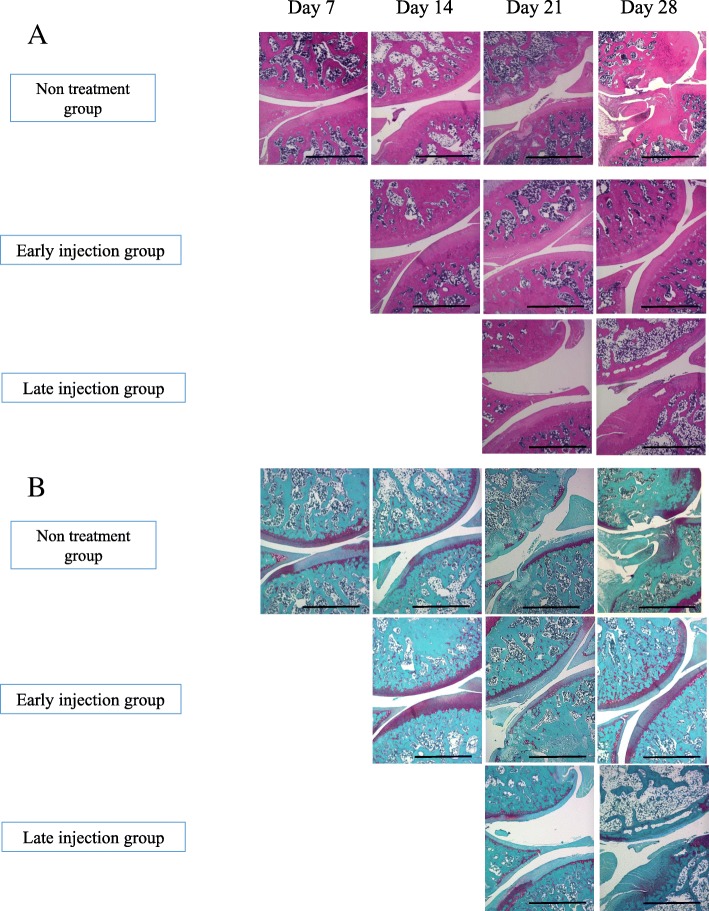
Histopathological analysis of the femoral and tibial condyles. Hematoxylin and eosin stain (**a**) and safranin-O staining (**b**) showed less cartilage degradation in the early injection group at days 14, 21, 28 after MIA injection, while the non-treatment group and the late injection group exhibited reduced cartilage thickness and superficial cartilage erosion

In the untreated OA group, cartilage erosion was observed at 2 weeks, and bone destruction became evident at 3 weeks with further progression at 4 weeks (Fig. 3a). The knee joint scores at 7, 14, 21, and 28 days after MIA injection were 1.2, 2.8, 4.8, and 4.9, respectively. In addition, in rats of the late injection group, cartilage erosion was observed at the same time as the untreated OA group. The scores were 4.4 and 4.3 at 21 and 28 days after injection, respectively. In the early injection group, the cartilage surface was still glossy at 4 weeks. The knee joint scores were 1.2, 1.4, and 1.5 at 14, 21, and 28 days after MIA injection, respectively. The scores were lower in the treated knees of the early injection group, relative to the control knees (*P* < 0.05) (Fig. 3b). At 14 and 21 days after MIA injection, safranin-O staining of the treated knee joints of the early injection group showed less cartilage degradation (including surface aggregation of chondrocytes, fissuring, and reduced matrix staining), compared with the untreated OA group and the late injection group (which exhibited reduced cartilage thickness and superficial erosions) (Fig. 4).

### Effects of intraarticular injection of PLA cells on DRG

**Fig. 5:**
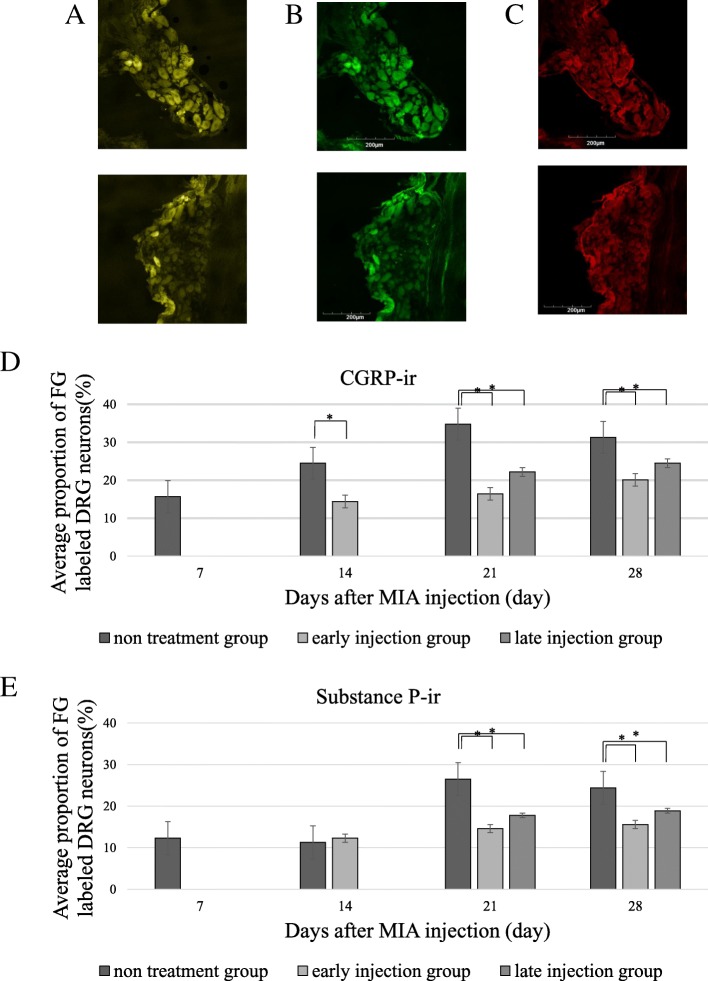
Representative fluorescence photomicrographs of DRG neurons. **a** FG-labeled DRG neurons, **b** CGRP-ir DRG neurons, **c** Substance P- ir DRG neurons. All photomicrographs are from the same section. Average proportion of FG-labeled CGRP-ir (**d**) and Substance P- ir (**e**) DRG neurons. Note the significantly higher numbers of FG-labeled CGRP-ir and Substance P- ir DRG neurons at 21 and 28 days after intraarticular injection in the untreated OA group, whereas the DRG showed no significant changes in the expression of CGRP and Substance P in the late and early injection groups. Data are mean ± SD, *P* < 0.05, by one-way ANOVA, with Tukey’s posy-hoc analysis

**Fig. 6:**
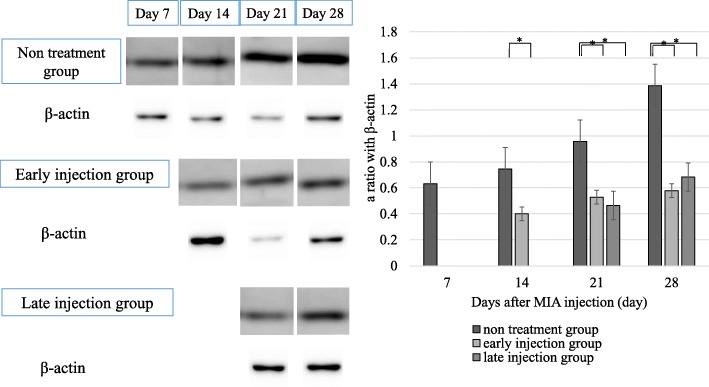
High expression levels of CGRP protein in DRG of the untreated OA group at 21 and 28 days after intraarticular injection, compared with slight increase in CGRP expression in the late and early injection groups. Data are mean ± SD, *P* < 0.05, by one-way ANOVA, with Tukey’s posy-hoc analysis

Examination of DRG demonstrated the presence of FG-labeled DRG neurons at all levels bilaterally. These neurons are known to innervate the knee joints. Furthermore, each specimen contained DRG neurons of all sizes (small, intermediate, and large), which were counted, together with the small size DRG neurons labeled with FG (Fig. 5a). Both CGRP-immunoreactive (−ir) (Fig. 5b) and substance P-ir (Fig. 5c) DRG neurons, representing pain-related biomarkers innervating the knee joint, were counted using fluorescence microscopy. Significantly higher expression levels of CGRP-ir (Fig. 5d) and substance P-ir (Fig. 5e) were detected in DRG of the untreated OA and late injection group at 21 and 28 days after the injection, compared with the early injection group (*P* < 0.05). Western blot analysis showed significantly higher relative expression levels of CGRP protein in DRG in the untreated OA group compared with the treatment groups (Fig. 6).

### Effects of intraarticular injection of mesenchymal stem cells on synovium

**Fig. 7:**
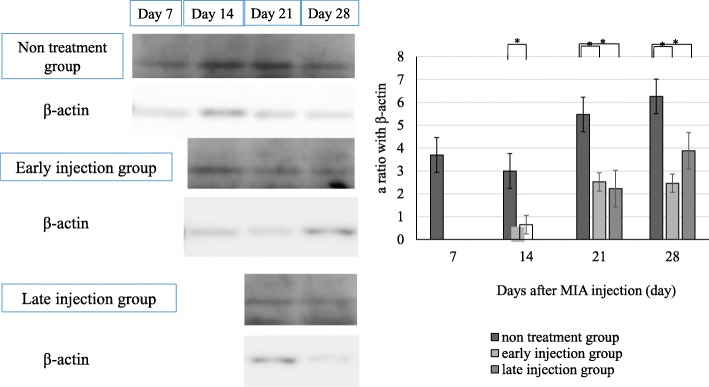
Low TNF-α protein expression levels in the joint capsule and synovium of the treatment groups compared with that in the synovia of untreated OA group. Data are mean ± SD, *P* < 0.05, by one-way ANOVA, with Tukey’s posy-hoc analysis

Western blot analysis showed significantly lower relative expression levels of TNF-α protein in the synovia of the treatment groups than the untreated OA group (Fig. 7).

## Discussion

The aim of the present study was to evaluate the therapeutic effects (cartilage repair/regeneration, pain, inflammatory change) of allogeneic PLA cells injected directly into the knee joints of rats with mild and severe experimentally-induced knee OA. For this purpose, we conducted the following procedures. First, macroscopic evaluation of the joint cartilage using gross score and EMS. Second, evaluation of pain of arthritis using immunohistochemistry and western blotting of SP, CGRP in DRG. Third, evaluation of TNF-α as an inflammatory cytokine, in the synovium of arthritis joint. The results demonstrated that direct intraarticular injection of allogeneic PLA cells into the joints of rodent knee OA model induced by MIA resulted in regeneration of the articular cartilage in rats of the early injection group, but not in those of the late injection group. Interestingly, cartilage regeneration and pain reduction were observed after treatment with PLA cells in both the early and late injection groups. This is the first report that compares the therapeutic effects of direct intraarticular injection of PLA cells on cartilage degeneration and pain reduction in MIA-induced knee OA.

MSCs therapy has yielded encouraging results in experimental models of knee OA [21–23]. These experimental studies have suggested that MSCs can halt cartilage degeneration in knee OA. A few clinical studies have also investigated the effects of MSCs in knee OA, and their results suggest that MSCs can reduce pain and improve function [24]. In the present study, we evaluated the effect of intraarticular injection of PLA cells at 7 and 14 days after MIA injection in rats. In both the early and late injection group, immunohistochemical examination showed downregulation of CGRP-ir and substance P-ir in the DRG neurons. On the other hand, in the untreated OA group and the late injection group, histopathological examination showed reduction of cartilage thickness, loss of cartilage matrix, and superficial cartilage erosion in the knee joints; although these changes were less marked in the early injection group. Similar results were found in the knee scores, with significant differences between the early injection group and the other groups. Our results indicate that intraarticular injection of PLA cells reduces pain and seems to be therapeutically beneficial in early-stage rodent OA.

Pre-clinical studies have generally focused on joint damage, although pain is a common symptom in clinical practice. Because it is difficult to assess the effect of PLA cells on pain in animal studies, there is little or no information on this sensory modality in experimental research studies. The MIA model has been used in several studies as an animal model of pain in OA [25, 26] and is suitable for studying this important OA-related sensation. Pain sensation is a complex process, and different pain types have been described together with different etiomechanisms, including inflammatory and neuropathic mechanisms. Pain can be evaluated indirectly in animal studies (e.g.*,* by analysis of weight bearing or gait) or can be assessed directly (e.g.*,* mechanical or thermal stimulation followed by paw withdrawal response). Regardless of the method used, it is relatively difficult to evaluate the results of pain assessment in animal experiments. Previous experimental studies focused on DRG to investigate the mechanisms of inflammation-related pain. Functionally, DRG neurons can be classified into three different types; i) large neurons, which are involved in proprioception [27], ii) small neurons with axon terminals containing among other neurochemicals, both substance P and CGRP, two pain-related neuropeptides [28–30], and iii) small neurons containing isolectin-B4 (IB4, a structural protein of *Griffonia simplicifolia*) in their synapses but lack substance P and CGRP. CGRP is known to cause hyperalgesia, and high levels of CGRP are thought to be involved in inflammation-related pain [31, 32]. Intraarticular injection of MIA was reported to induce significant up-regulation of substance P and CGRP mRNA expression in DRG neurons, as well as a significant increase in substance P and CGRP immunoreactivity in the synovium, periosteum, and subchondral bone, compared to the control [33]. Based on this background, we evaluated pain in our animal model by measuring substance P- and CGRP-positive DRG neurons.

The present study had certain limitations. First, MIA-induced OA is a model of chemical OA. Therefore, further investigation should be conducted in other nonchemical OA models, such as Hartley guinea pigs that spontaneously develop cartilage degeneration in the knee joints. Second, the samples from each knee contained both the synovium and joint capsule, but we did not map the localization of proinflammatory cytokines and PLA cells in each tissue component. Furthermore, we evaluated the analgesic effect of the treatment using only a limited number of proinflammatory cytokines and without assessment of pain behavior. Third, we examined changes up to 4 weeks after MIA injection. Because neuropathic pain tends to increase over time, further long-term studies are needed to assess the profile of OA-related inflammatory pain affecting the knee.

In this study, the articular cartilage of the early injection group was examined serially for up to 28 days after the injection of PLA cells. The described changes were observed throughout this period and our results showed no differences among the different time points of assessment in these animals. Furthermore, our results showed no significant differences in the macroscopic score between mice of the no treatment group and those of the late injection group. Does this finding mean that PLA cells containing AD-MSCs injected directly into the knee joint have no or very little capacity for regeneration of the degenerative cartilages? Delanois and coworkers [33] reviewed the outcome of AD-MSCs injection in knee OA and concluded that they have anti-inflammatory and analgesic effects but do not improve degenerative changes in human knee OA. Our results were similar to their findings and conclusion. Interestingly, the findings of an increasing number of articles on basic research, animal experiments, and clinical trials provide support to the therapeutic effects of MSCs in OA. Admittedly, many issues remain uncertain with respect to the therapeutic benefits of MSCs in OA. For example, the mechanisms of action in OA, immune issues associated with allogeneic transplantation, and precautions regarding culture/expansion of these cells [24, 34]. Injection of MSCs encapsulated in self-assembled peptide hydrogels into the articular cavity in an OA rat model provided chondroprotection and resulted in downregulation of several biomarkers of inflammation and apoptosis [35]. MSCs inhibited the progression of cartilage degeneration by homing to the synovium and secreting a liquid factor with chondro-protective effects, such as chondrocyte proliferation and cartilage matrix protection [36]. Our findings also support the use of PLA cells in OA. However, several issues require further investigation, including the mechanism of PLA cells implantation, mechanism of the anti-inflammatory activity of PLA cells, and the process of PLA cells differentiation. We will continue to focus on these problems in future studies.

## Conclusions

The major findings of the present study were *1)* Direct intraarticular injection of PLA cells prevented degenerative changes in the early injection group but had weak effect on cartilage repair in the late injection group. *2)* Injection of PLA cells resulted in comparable level of downregulation of expression of SP and CGRP at DRG in the early and late injection groups. In other words, direct intraarticular injection of PLA cells in the knee OA joint had pain suppressive effect approximately equally in both the early and late injection groups. *3)* Direct intraarticular injection of PLA cells was associated with downregulation of TNF-α in the synovium of knee OA joints.

## Data Availability

The datasets used and/or analyzed during the study are available from the corresponding author upon request.

## Abbreviations

AD-MSCs: Adipose derived-mesenchymal stem cells
CGRP: Calcitonin gene-related peptide
DMEM: Dulbecco’s modified Eagle’s medium
DRG: Dorsal root ganglion
EDTA: Ethylenediaminetetraacetic acid
FBS: Fetal bovine serum
K/L grade: Kellgren and Lawrence grade
MIA: Monoiodoacetate
MSCs: Mesenchymal stem cells
OA: Osteoarthritis
PBS: Phosphate-buffered saline
PLA: Processed lipoaspirate
SP: Substance P
